# Supplementary material for: Discovery and application of insertion-deletion (INDEL) polymorphisms for QTL mapping of early life-history traits in Atlantic salmon
Source: BMC Genomics. 2010 Mar 8;11:156. doi: 10.1186/1471-2164-11-156 (PMC2838853; doi:10.1186/1471-2164-11-156)
Supplement: Additional file 2 — Information on developed 76 locus single-run INDEL panel in Atlantic salmon. Information on fluorescence labeling, primer concentrations, PCR pooling and links to alignments, INDEL motifs and GENESCAN (Burge and Karlin 1997) predictions of genes/exons are available in html format. [file 1471-2164-11-156-S2.ZIP › Additionalfile2/table4493.html]

Contig Alignment

```
Base	A B C D E F G H I J K L M N O P Q   
1	C . . . . . . . . . . . . . . . .    
2	A . . . . . . . . . . . . . . . .    
3	G . . . . . . . . . . . . . . . .    
4	G . . . . . . . . . . . . . . . .    
5	C . . . . . . . . . . . . . . . .    
6	C . . . . . . . . . . . . . . . .    
7	A . . . . . . . . . . . . . . . .    
8	C . . . . . . . . . . . . . . . .    
9	A . . . . . . . . . . . . . . . .    
10	C . . . . . . . . . . . . . . . .    
11	A . . . . . . . . . . . . . . . .    
12	T . . . . . . . . . . . . . . . .    
13	T . . . . . . . . . . . . . . . .    
14	T . . . . . . . . . . . . . . . .    
15	T . . . . . . . . . . . . . . . .    
16	A . . . . . . . . . . . . . . . .    
17	A . . . . . . . . . . . . . . . .    
18	T . . . . . . . . . . . . . . . .    
19	T . . . . . . . . . . . . . . . .    
20	C . . . . . . . . . . . . . . . .    
21	A . . . . . . . . . . . . . . . .    
22	C . . . . . . . . . . . . . . . .    
23	A . . . . . . . . . . . . . . . .    
24	G . . . . . . . . . . . . . . . .    
25	G . . . . . . . . . . . . . . . .    
26	C . . . . . . . . . . . . . . . .    
27	A . . . . . . . . . . . . . . . .    
28	A . . . . . . . . . . . . . . . .    
29	T . . . . . . . . . . . . . . . .    
30	A . . . . . . . . . . . . . . . .    
31	A . . . . . . . . . . . . . . . .    
32	A . . . . . . . . . . . . . . . .    
33	G . . . . . . . . . . . . . . . .    
34	A . . . . . . . . . . . . . . . .    
35	T . . . . . . . . . . . . . . . .    
36	A . . . . . . . . . . . . . . . .    
37	A . . . . . . . . . . . . . . . .    
38	A . . . . . . . . . . . . . . . .    
39	A . . . . . . . . . . . . . . . .    
40	C . . . . . . . . . . . . . . . .    
41	A . . . . . . . . . . . . . . . .    
42	T . . . . . . . . . . . . . . . .    
43	C . . . . . . . . . . . . . . . .    
44	A . . . . . . . . . . . . . . . .    
45	G . . . . . . . . . . . . . . . .    
46	G . . . . . . . . . . . . . . . .    
47	T . . . . . . . . . . . . . . . .    
48	A . . . . . . . . . . . . . . . .    
49	A . . . . . . . . . . . . . . . .    
50	A . . . . . . . . . . . . . . . .    
51	A . . . . . . . . . . . . . . . .    
52	A . . . . . . . . . . . . . . . .    
53	A . . . . . . . . . . . . . . . .    
54	C . . . . . . . . . . . . . . . .    
55	C . . . . . . . . . . . . . . . .    
56	T . . . . . . . . . . . . . . . .    
57	A . . . . . . . . . . . . . . . .    
58	G . . . . . . . . . . . . . . . .    
59	G . . . . . . . . . . . . . . . .    
60	C . . . . . . . . . . . . . . . .    
61	T . . . . . . . . . . . . . . . .    
62	T . . . . . . . . . . . . . . . .    
63	C . . . . . . . . . . . . . . . .    
64	A . . . . . . . . . . . . . . . .    
65	T . . . . . . . . . . . . . . . .    
66	T . . . . . . . . . . . . . . . .    
67	T . . . . . . . . . . . . . . . .    
68	T . . . . . . . . . . . . . . . .    
69	A . . . . . . . . . . . . . . . .    
70	G . . . . . . . . . . . . . . . .    
71	A . . . . . . . . . . . . . . . .    
72	T . . . . . . . . . . . . . . . .    
73	T . . . . . . . . . . . . . . . .    
74	C . . . . . . . . . . . . . . . .    
75	T . . . . . . . . . . . . . . . .    
76	G . . . . . . . . . . . . . . . .    
77	A . . . . . . . . . . . . . . . .    
78	A . . . . . . . . . . . . . . . .    
79	C . . . . . . . . . . . . . . . .    
80	T . . . . . . . . . . . . . . . .    
81	C . . . . . . . . . . . . . . . .    
82	A . . . . . . . . . . . . . . . .    
83	A . . . . . . . . . . . . . . . .    
84	T . . . . . . . . . . . . . . . .    
85	T . . . . . . . . . . . . . . . .    
86	T . . . . . . . . . . . . . . . .    
87	C . . . . . . . . . . . . . . . .    
88	G . . . . . . . . . . . . . . . .    
89	A . . . . . . . . . . . . . . . .    
90	A . . . . . . . . . . . . . . . .    
91	T . . . . . . . . . . . . . . . .    
92	C . . . . . . . . . . . . . . . .    
93	A . . . . . . . . . . . . . . . .    
94	C . . . . . . . . . . . . . . . .    
95	A . . . . . . . . . . . . . . . .    
96	C . . . . . . . . . . . . . . . .    
97	A . . . . . . . . . . . . . . . .    
98	T . . . . . . . . . . . . . . . .    
99	T . . . . . . . . . . . . . . . .    
100	A . . . . . . . . . . . . . . . .    
101	G . . . . . . . . . . . . . . . .    
102	G . . . . . . . . . . . . . . . .    
103	A . . . . . . . . . . . . . . . .    
104	A . . . . . . . . . . . . . . . .    
105	T . . . . . . . . . . . . . . . .    
106	G . . . . . . . . . . . . . . . .    
107	T . . . . . . . . . . . . . . . .    
108	G . . . . . . . . . . . . . . . .    
109	A . . . . . . . . . . . . . . . .    
110	C . . . . . . . . . . . . . . . .    
111	C . . . . . . . . . . . . . . . .    
112	A . . . . . . . . . . . . . . . .    
113	A . . . . . . . . . . . . . . . .    
114	A . . . . . . . . . . . . . . . .    
115	A . . . . . . . . . . . . . . . .    
116	T . . . . . . . . . . . . . . . .    
117	A . . . . . . . . . . . . . . . .    
118	G . . . . . . . . . . . . . . . .    
119	C . . . . . . . . . . . . . . . .    
120	T . . . . . . . . . . . . . . . .    
121	T . . . . . . . . . . . . . . . .    
122	T . . . . . . . . . . . . . . . .    
123	T . . . . . . . . . . . . . . . .    
124	T . . . . . . . . . . . . . . . .    
125	A . . . . . . . . . . . . . . . .    
126	C . . . . . . . . . . . . . . . .    
127	T . . . . . . . . . . . . . . . .    
128	A . . . . . . . . . . . . . . . .    
129	C . . . . . . . . . . . . . . . .    
130	C . . . . . . . . . . . . . . . .    
131	T . . . . . . . . . . . . . . . .    
132	G . . . . . . . . . . . . . . . .    
133	A . . . . . . . . . . . . . . . .    
134	G . . . . . . . . . . . . . . . .    
135	G . . . . . . . . . . . . . . . .    
136	A . . . . . . . . . . . . . . . .    
137	A . . . . . . . . . . . . . . . .    
138	C . . . . . . . . . . . . . . . .    
139	A . . . . . . . . . . . . . . . .    
140	T . . . . . . . . . . . . . . . .    
141	T . . . . . . . . . . . . . . . .    
142	G . . . . . . . . . . . . . . . .    
143	C . . . . . . . . . . . . . . . .    
144	C . . . . . . . . . . . . . . . .    
145	A . . . . . . . . . . . . . . . .    
146	A . . . . . . . . . . . . . . . .    
147	G . . . . . . . . . . . . . . . .    
148	G . . . . . . . . . . . . . . . .    
149	T . . . . . . . . . . . . . . . .    
150	G . . . . . . . . . . . . . . . .    
151	T . . . . . . . . . . . . . . . .    
152	G . . . . . . . . . . . . . . . .    
153	G . . . . . . . . . . . . . . . .    
154	C . . . . . . . . . . . . . . . .    
155	C . . . . . . . . . . . . . . . .    
156	G . . . . . . . . . . . . . . . .    
157	T . . . . . . . . . . . . . . . .    
158	T . . . . . . . . . . . . . . . .    
159	T . . . . . . . . . . . . . . . .    
160	C . . . . . . . . . . . . . . . .    
161	T . . . . . . . . . . . . . . . .    
162	C . . . . . . . . . . . . . . . .    
163	T . . . . . . . . . . . . . . . .    
164	C . . . . . . . . . . . . . . . .    
165	G . . . . . . . . . . . . . . . .    
166	C . . . . . . . . . . . . . . . .    
167	A . . . . . . . . . . . . . . . .    
168	G . . . . . . . . . . . . . . . .    
169	G . . . . . . . . . . . . . . . .    
170	C . . . . . . . . . . . . . . . .    
171	T . . . . . . . . . . . . . . . .    
172	A . . . . . . . . . . . . . . . .    
173	A . . . . . . . . . . . . . . . .    
174	T . . . . . . . . . . . . . . . .    
175	A . . . . . . . . . . . . . . . .    
176	C . . . . . . . . . . . . . . . .    
177	A . . . . . . . . . . . . . . . .    
178	T . . . . . . . . . . . . . . . .    
179	A . . . . . . . . . . . . . . . .    
180	A . . . . . . . . . . . . . . . .    
181	A . . . . . . . . . . . . . . . .    
182	G . . . . . . . . . . . . . . . .    
183	A . . . . . . . . . . . . . . . .    
184	T . . . . . . . . . . . . . . . .    
185	T . . . . . . . . . . . . . . . .    
186	C . . . . . . . . . . . . . . . .    
187	A . . . . . . . . . . . . . . . .    
188	T . . . . . . . . . . . . . . . .    
189	C . . . . . . . . . . . . . . . .    
190	C . . . . . . . . . . . . . . . .    
191	A . . . . . . . . . . . . . . . .    
192	T . . . . . . . . . . . . . . . .    
193	G . . . . . . . . . . . . . . . .    
194	C . . . . . . . . . . . . . . . .    
195	T . . . . . . . . . . . . . . . .    
196	T . . . . . . . . . . . . . . . .    
197	T . . . . . . . . . . . . . . . .    
198	T . . . . . . . . . . . . . . . .    
199	A . . . . . . . . . . . . . . . .    
200	T . . . . . . . . . . . . . . . .    
201	T . . . . . . . . . . . . . . . .    
202	A . . . . . . . . . . . . . . . .    
203	C . . . . . . . . . . . . . . . .    
204	A . . . . . . . . . . . . . . . .    
205	A . . . . . . . . . . . . . . . .    
206	G . . . . . . . . . . . . . . . .    
207	C . . . . . . . . . . . . . . . .    
208	A . . . . . . . . . . . . . . . .    
209	A . . . . . . . . . . . . . . . .    
210	G . . . . . . . . . . . . . . . .    
211	C . . . . . . . . . . . . . . . .    
212	T . . . . . . . . . . . . . . . .    
213	T . . . . . . . . . . . . . . . .    
214	G . . . . . . . . . . . . . . . .    
215	A . . . . . . . . . . . . . . . .    
216	C . . . . . . . . . . . . . . . .    
217	T . . . . . . . . . . . . . . . .    
218	A . . . . . . . . . . . . . . . .    
219	C . . . . . . . . . . . . . . . .    
220	T . . . . . . . . . . . . . . . .    
221	G . . . . . . . . . . . . . . . .    
222	T . . . . . . . . . . . . . . . .    
223	A . . . . . . . . . . . . . . . .    
224	A . . . . . . . . . . . . . . . .    
225	T . . . . . . . . . . . . . . . .    
226	G . . . . . . . . . . . . . . . .    
227	T . . . . . . . . . . . . . . . .    
228	T . . . . . . . . . . . . . . . .    
229	C . . . . . . . . . . . . . . . .    
230	T . . . . . . . . . . . . . . . .    
231	C . . . . . . . . . . . . . . . .    
232	C . . . . . . . . . . . . . . . .    
233	T . . . . . . . . . . . . . . . .    
234	G . . . . . . . . . . . . . . . .    
235	G . . . . . . . . . . . . . . . .    
236	C . . . . . . . . . . . . . . . .    
237	T . . . . . . . . . . . . . . . .    
238	G . . . . . . . . . . . . . . . .    
239	G . . . . . . . . . . . . . . . .    
240	T . . . . . . . . . . . . . . . .    
241	C . . . . . . . . . . . . . . . .    
242	T . . . . . . . . . . . . . . . .    
243	G . . . . . . . . . . . . . . . .    
244	G . . . . . . . . . . . . . . . .    
245	T . . . . . . . . . . . . . . . .    
246	C . . . . . . . . . . . . . . . .    
247	T . . . . . . . . . . . . . . . .    
248	G . . . . . . . . . . . . . . . .    
249	C . . . . . . . . . . . . . . . .    
250	C . . . . . . . . . . . . . . . .    
251	C . . . . . . . . . . . . . . . .    
252	A . . . . . . . . . . . . . . . .    
253	A . . . . . . . . . . . . . . . .    
254	G . . . . . . . . . . . . . . . .    
255	A . . . . . . . . . . . . . . . .    
256	A . . . . . . . . . . . . . . . .    
257	A . . . . . . . . . . . . . . . .    
258	G . . . . . . . . . . . . . . . .    
259	C . . . . . . . . . . . . . . . .    
260	C . . . . . . . . . . . . . . . .    
261	A . . . . . . . . . . . . . . . .    
262	T . . . . . . . . . . . . . . . .    
263	T . . . . . . . . . . . . . . . .    
264	G . . . . . . . . . . . . . . . .    
265	G . . . . . . . . . . . . . . . .    
266	T . . . . . . . . . . . . . . . .    
267	C . . . . . . . . . . . . . . . .    
268	A . . . . . . . . . . . . . . . .    
269	A . . . . . . . . . . . . . . . .    
270	C . . . . . . . . . . . . . . . .    
271	A . . . . . . . . . . . . . . . .    
272	G . . . . . . . . . . . . . . . .    
273	C . . . . . . . . . . . . . . . .    
274	T . . . . . . . . . . . . . . . .    
275	A . . . . . . . . . . . . . . . .    
276	A . . . . . . . . . . . . . . . .    
277	A . . . . . . . . . . . . . . . .    
278	C . . . . . . . . . . . . . . . .    
279	A . . . . . . . . . . . . . . . .    
280	T . . . . . . . . . . . . . . . .    
281	A . . . . . . . . . . . . . . . .    
282	C . . . . . . . . . . . . . . . .    
283	A . . . . . . . . . . . . . . . .    
284	G . . . . . . . . . . . . . . . .    
285	A . . . . . . . . . . . . . . . .    
286	A . . . . . . . . . . . . . . . .    
287	T . . . . . . . . . . . . . . . .    
288	G . . . . . . . . . . . . . . . .    
289	C . . . . . . . . . . . . . . . .    
290	T . . . . . . . . . . . . . . . .    
291	G . . . . . . . . . . . . . . . .    
292	C . . . . . . . . . . . . . . . .    
293	A . . . . . . . . . . . . . . . .    
294	G . . . . . . . . . . . . . . . .    
295	C . . . . . . . . . . . . . . . .    
296	A . . . . . . . . . . . . . . . .    
297	C . . . . . . . . . . . . . . . .    
298	G . . . . . . . . . . . . . . . .    
299	G . . . . . . . . . . . . . . . .    
300	G . . . . . . . . . . . . . . . .    
301	T . . . . . . . . . . . . . . . .    
302	A . . . . . . . . . . . . . . . .    
303	C . . . . . . . . . . . . . . . .    
304	T . . . . . . . . . . . . . . . .    
305	G . . . . . . . . . . . . . . . .    
306	A . . . . . . . . . . . . . . . .    
307	C . . . . . . . . . . . . . . . .    
308	C . . . . . . . . . . . . . . . .    
309	A . . . . . . . . . . . . . . . .    
310	A . . . . . . . . . . . . . . . .    
311	G . . . . . . . . . . . . . . . .    
312	A . . . . . . . . . . . . . . . .    
313	C . . . . . . . . . . . . . . . .    
314	C . . . . . . . . . . . . . . . .    
315	A . . . . . . . . . . . . . . . .    
316	G . . . . . . . . . . . . . . . .    
317	A . . . . . . . . . . . . . . . .    
318	G . . . . . . . . . . . . . . . .    
319	G . . . . . . . . . . . . . . . .    
320	G . . . . . . . . . . . . . . . .    
321	A . . . . . . . . . . . . . . . .    
322	G . . . . . . . . . . . . . . . .    
323	A . . . . . . . . . . . . . . . .    
324	G . . . . . . . . . . . . . . . .    
325	C . . . . . . . . . . . . . . . .    
326	A . . . . . . . . . . . . . . . .    
327	C . . . . . . . . . . . . . . . .    
328	A . . . . . . . . . . . . . . . .    
329	T . . . . . . . . . . . . . . . .    
330	A . . . . . . . . . . . . . . . .    
331	T . . . . . . . . . . . . . . . .    
332	T . . . . . . . . . . . . . . . .    
333	A . . . . . . . . . . . . . . . .    
334	C . . . . . . . . . . . . . . . .    
335	A . . . . . . . . . . . . . . . .    
336	C . . . . . . . . . . . . . . . .    
337	A . . . . . . . . . . . . . . . .    
338	G . . . . . . . . . . . . . . . .    
339	G . . . . . . . . . . . . . . . .    
340	T . . . . . . . . . . . . . . . .    
341	C . . . . . . . . . . . . . . . .    
342	A . . . . . . . . . . . . . . . .    
343	T . . . . . . . . . . . . . . . .    
344	A . . . . . . . . . . . . . . . .    
345	A . . . . . . . . . . . . . . . .    
346	G . . . . . . . . . . . . . . . .    
347	G . . . . . . . . . . . . . . . .    
348	T . . . . . . . . . . . . . . . .    
349	C . . . . . . . . . . . . . . . .    
350	T . . . . . . . . . . . . . . . .    
351	C . . . . . . . . . . . . . . . .    
352	T . . . . . . . . . . . . . . . .    
353	G . . . . . . . . . . . . . . . .    
354	C . . . . . . . . . . . . . . . .    
355	A . . . . . . . . . . . . . . . .    
356	C . . . . . . . . . . . . . . . .    
357	T . . . . . . . . . . . . . . . .    
358	G . . . . . . . . . . . . . . . .    
359	G . . . . . . . . . . . . . . . .    
360	C . . . . . . . . . . . . . . . .    
361	T . . . . . . . . . . . . . . . .    
362	G . . . . . . . . . . . . . . . .    
363	C . . . . . . . . . . . . . . . .    
364	C . . . . . . . . . . . . . . . .    
365	T . . . . . . . . . . . . . . . .    
366	G . . . . . . . . . . . . . . . .    
367	T . . . . . . . . . . . . . . . .    
368	G . . . . . . . . . . . . . . . .    
369	A . . . . . . . . . . . . . . . .    
370	G . . . . . . . . . . . . . . . .    
371	C . . . . . . . . . . . . . . . .    
372	T . . . . . . . . . . . . . . . .    
373	G . . . . . . . . . . . . . . . .    
374	T . . . . . . . . . . . . . . . .    
375	G . . . . . . . . . . . . . . . .    
376	G . . . . . . . . . . . . . . . .    
377	A . . . . . . . . . . . . . . . .    
378	A . . . . . . . . . . . . . . . .    
379	T . . . . . . . . . . . . . . . .    
380	T . . . . . . . . . . . . . . . .    
381	A . . . . . . . . . . . . . . . .    
382	A . . . . . . . . . . . . . . . .    
383	T . . . . . . . . . . . . . . . .    
384	G . . . . . . . . . . . . . . . .    
385	T . . . . . . . . . . . . . . . .    
386	T . . . . . . . . . . . . . . . .    
387	A . . . . . . . . . . . . . . . .    
388	A . . . . . . . . . . . . . . . .    
389	G . . . . . . . . . . . . . . . .    
390	A . . . . . . . . . . . . . . . .    
391	T . . . . . . . . . . . . . . . .    
392	T . . . . . . . . . . . . . . . .    
393	C . . . . . . . . . . . . . . . .    
394	T . . . . . . . . . . . . . . . .    
395	T . . . . . . . . . . . . . . . .    
396	C . . . . . . . . . . . . . . . .    
397	T . . . . . . . . . . . . . . . .    
398	A . . . . . . . . . . . . . . . .    
399	T . . . . . . . . . . . . . . . .    
400	T . . . . . . . . . . . . . . . .    
401	G . . . . . . . . . . . . . . . .    
402	G . . . . . . . . . . . . . . . .    
403	T . . . . . . . . . . . . . . . .    
404	T . . . . . . . . . . . . . . . .    
405	T . . . . . . . . . . . . . . . .    
406	T . . . . . . . . . . . . . . . .    
407	T . . . . . . . . . . . . . . . .    
408	A . . . . . . . . . . . . . . . .    
409	A . . . . . . . . . . . . . . . .    
410	A . . . . . . . . . . . . . . . .    
411	T . . . . . . . . . . . . . . . .    
412	C . . . . . . . . . . . . . . . .    
413	A . . . . . . . . . . . . . . . .    
414	A . . . . . . . . . . . . . . . .    
415	T . . . . . . . . . . . . . . . .    
416	C . . . . . . . . . . . . . . . .    
417	C . . . . . . . . . . . . . . . .    
418	A . . . . . . . . . . . . . . . .    
419	C . . . . . . . . . . . . . . . .    
420	G . . . . . . . . . . . . . . . .    
421	A . . . . . . . . . . . . . . . .    
422	T . . . . . . . . . . . . . . . .    
423	T . . . . . . . . . . . . . . . .    
424	G . . . . . . . . . . . . . . . .    
425	T . . . . . . . . . . . . . . . .    
426	G . . . . . . . . . . . . . . . .    
427	C . . . . . . . . . . . . . . . .    
428	A . . . . . . . . . . . . . . . .    
429	C . . . . . . . . . . . . . . . .    
430	T . . . . . . . . . . . . . . . .    
431	C . . . . . . . . . . . . . . . .    
432	C . . . . . . . . . . . . . . . .    
433	A . . . . . . . . . . . . . . . .    
434	A . . . . . . . . . . . . . . . .    
435	T . . . . . . . . . . . . . . . .    
436	T . . . . . . . . . . . . . . . .    
437	C . . . . . . . . . . . . . . . .    
438	A . . . . . . . . . . . . . . . .    
439	T . . . . . . . . . . . . . . . .    
440	G . . . . . . . . . . . . . . . .    
441	T . . . . . . . . . . . . . . . .    
442	C . . . . . . . . . . . . . . . .    
443	A . . . . . . . . . . . . . . . .    
444	G . . . . . . . . . . . . . . . .    
445	A . . . . . . . . . . . . . . . .    
446	C . . . . . . . . . . . . . . . .    
447	A . . . . . . . . . . . . . . . .    
448	T . . . . . . . . . . . . . . . .    
449	A . . . . . . . . . . . . . . . .    
450	C . . . . . . . . . . . . . . . .    
451	T . . . . . . . . . . . . . . . .    
452	T . . . . . . . . . . . . . . . .    
453	T . . . . . . . . . . . . . . . .    
454	T . . . . . . . . . . . . . . . .    
455	A . . . . . . . . . . . . . . . .    
456	A . . . . . . . . . . . . . . . .    
457	G . . . . . . . . . . . . . . . .    
458	T . . . . . . . . . . . . . . . .    
459	T . . . . . . . . . . . . . . . .    
460	A . . . . . . . . . . . . . . . .    
461	T . . . . . . . . . . . . . . . .    
462	G . . . . . . . . . . . . . . . .    
463	T . . . . . . . . . . . . . . . .    
464	A . . . . . . . . . . . . . . . .    
465	C . . . . . . . . . . . . . . . .    
466	C . . . . . . . . . . . . . . . .    
467	C . . . . . . . . . . . . . . . .    
468	A . . . . . . . . . . . . . . . .    
469	G . . . . . . . . . . . . . . . .    
470	T . . . . . . . . . . . . . . . .    
471	A . . . . . . . . . . . . . . . .    
472	G . . . . . . . . . . . . . . . .    
473	A . . . . . . . . . . . . . . . .    
474	T . . . . . . . . . . . . . . . .    
475	C . . . . . . . . . . . . . . . .    
476	C . . . . . . . . . . . . . . . .    
477	C . . . . . . . . . . . . . . . .    
478	T . . . . . . . . . . . . . . . .    
479	C . . . . . . . . . . . . . . . .    
480	A . . . . . . . . . . . . . . . .    
481	G . . . . . . . . . . . . . . . .    
482	G . . . . . . . . . . . . . . . .    
483	T . . . . . . . . . . . . . . . .    
484	C . . . . . . . . . . . . . . . .    
485	C . . . . . . . . . . . . . . . .    
486	T . . . . . . . . . . . . . . . .    
487	C . . . . . . . . . . . . . . . .    
488	T . . . . . . . . . . . . . . . .    
489	G . . . . . . . . . . . . . . . .    
490	G . . . . . . . . . . . . . . . .    
491	C . . . . . . . . . . . . . . . .    
492	A . . . . . . . . . . . . . . . .    
493	C . . . . . . . . . . . . . . . .    
494	T . . . . . . . . . . . . . . . .    
495	G . . . . . . . . . . . . . . . .    
496	G . . . . . . . . . . . . . . . .    
497	C . . . . . . . . . . . . . . . .    
498	C . . . . . . . . . . . . . . . .    
499	T . . . . . . . . . . . . . . . .    
500	T . . . . . . . . . . . . . . . .    
501	T . . . . . . . . . . . . . . . .    
502	T . . . . . . . . . . . . . . . .    
503	A . . . . . . . . . . . . . . . .    
504	A . . . . . . . . . . . . . . . .    
505	C . . . . . . . . . . . . . . . .    
506	T . . . . . . . . . . . . . . . .    
507	A . . . . . . . . . . . . . . . .    
508	T . . . . . . . . . . . . . . . .    
509	C . . . . . . . . . . . . . . . .    
510	C . . . . . . . . . . . . . . . .    
511	C . . . . . . . . . . . . . . . .    
512	A . . . . . . . . . . . . . . . .    
513	A . . . . . . . . . . . . . . . .    
514	A . . . . . . . . . . . . . . . .    
515	G . . . . . . . . . . . . . . . .    
516	C . . . . . . . . . . . . . . . .    
517	C . . . . . . . . . . . . . . . .    
518	T . . . . . . . . . . . . . . . .    
519	A . . . . . . . . . . . . . . . .    
520	G . . . . . . . . . . . . . . . .    
521	G . . . . . . . . . . . . . . . .    
522	A . . . . . . . . . . . . . . . .    
523	C . . . . . . . . . . . . . . . .    
524	C . . . . . . . . . . . . . . . .    
525	A . . . . . . . . . . . . . . . .    
526	A . . . . . . . . . . . . . . . .    
527	G . . . . . . . . . . . . . . . .    
528	A . . . . . . . . . . . . . . . .    
529	G . . . . . . . . . . . . . . . .    
530	G . . . . . . . . . . . . . . . .    
531	C . . . . . . . . . . . . . . . .    
532	A . . . . . . . . . . . . . . . .    
533	T . . . . . . . . . . . . . . . .    
534	G . . . . . . . . . . . . . . . .    
535	G . . . . . . . . . . . . . . . .    
536	A . . . . . . . . . . . . . . . .    
537	G . . . . . . . . . . . . . . . .    
538	A . . . . . . . . . . . . . . . .    
539	G . . . . . . . . . . . . . . . .    
540	G . . . . . . . . . . . . . . . .    
541	C . . . . . . . . . . . . . . . .    
542	A . . . . . . . . . . . . . . . .    
543	G . . . . . . . . . . . . . . . .    
544	C . . . . . . . . . . . . . . . .    
545	C . . . . . . . . . . . . . . . .    
546	T . . . . . . . . . . . . . . . .    
547	T . . . . . . . . . . . . . . . .    
548	C . . . . . . . . . . . . . . . .    
549	A . . . . . . . . . . . . . . . .    
550	G . . . . . . . . . . . . . . . .    
551	T . . . . . . . . . . . . . . . .    
552	T . . . . . . . . . . . . . . . .    
553	A . . . . . . . . . . . . . . . .    
554	A . . . . . . . . . . . . . . . .    
555	T . . . . . . . . . . . . . . . .    
556	A . . . . . . . . . . . . . . . .    
557	T . . . . . . . . . . . . . . . .    
558	G . . . . . . . . . . . . . . . .    
559	C . . . . . . . . . . . . . . . .    
560	C . . . . . . . . . . . . . . . .    
561	C . . . . . . . . . . . . . . . .    
562	C . . . . . . . . . . . . . . . .    
563	C . . . . . . . . . . . . . . . .    
564	A . . . . . . . . . . . . . . . .    
565	G . . . . . . . . . . . . . . . .    
566	C . . . . . . . . . . . . . . . .    
567	C . . . . . . . . . . . . . . . .    
568	T . . . . . . . . . . . . . . . .    
569	C . . . . . . . . . . . . . . . .    
570	T . . . . . . . . . . . . . . . .    
571	G . . . . . . . . . . . . . . . .    
572	G . . . . . . . . . . . . . . . .    
573	A . . . . . . . . . . . . . . . .    
574	A . . . . . . . . . . . . . . . .    
575	T . . . . . . . . . . . . . . . .    
576	A . . . . . . . . . . . . . . . .    
577	G . . . . . . . . . . . . . . . .    
578	C . . . . . . . . . . . . . . . .    
579	C . . . . . . . . . . . . . . . .    
580	T . . . . . . . . . . . . . . . .    
581	G . . . . . . . . . . . . . . . .    
582	C . . . . . . . . . . . . . . . .    
583	C . . . . . . . . . . . . . . . .    
584	A . . . . . . . . . . . . . . . .    
585	G . . . . . . . . . . . . . . . .    
586	A . . . . . . . . . . . . . . . .    
587	G . . . . . . . . . . . . . . . .    
588	A . . . . . . . . . . . . . . . .    
589	A . . . . . . . . . . . . . . . .    
590	C . . . . . . . . . . . . . . . .    
591	C . . . . . . . . . . . . . . . .    
592	C . . . . . . . . . . . . . . . .    
593	A . . . . . . . . . . . . . . . .    
594	A . . . . . . . . . . . . . . . .    
595	G . . . . . . . . . . . . . . . .    
596	G . . . . . . . . . . . . . . . .    
597	G . . . . . . . . . . . . . . . .    
598	G . . . . . . . . . . . . . . . .    
599	G . . . . . . . . . . . . . . . .    
600	G . . . . . . . . . . . . . . . .    
601	C . . . . . . . . . . . . . . . .    
602	C . . . . . . . . . . . . . . . .    
603	A . . . . . . . . . . . . . . . .    
604	A . . . . . . . . . . . . . . . .    
605	A . . . . . . . . . . . . . . . .    
606	A . . . . . . . . . . . . . . . .    
607	C . . . . . . . . . . . . . . . .    
608	T . . . . . . . . . . . . . . . .    
609	G . . . . . . . . . . . . . . . .    
610	T . . . . . . . . . . . . . . . .    
611	G . . . . . . . . . . . . . . . .    
612	G . . . . . . . . . . . . . . . .    
613	A . . . . . . . . . . . . . . . .    
614	C . . . . . . . . . . . . . . . .    
615	A . . . . . . . . . . . . . . . .    
616	T . . . . . . . . . . . . . . . .    
617	A . . . . . . . . . . . . . . . .    
618	T . . . . . . . . . . . . . . . .    
619	T . . . . . . . . . . . . . . . .    
620	T . . . . . . . . . . . . . . . .    
621	A . . . . . . . . . . . . . . . .    
622	A . . . . . . . . . . . . . . . .    
623	G . . . . . . . . . . . . . . . .    
624	A . . . . . . . . . . . . . . . .    
625	G . . . . . . . . . . . . . . . .    
626	A . . . . . . . . . . . . . . . .    
627	T . . . . . . . . . . . . . . . .    
628	C . . . . . . . . . . . . . . . .    
629	T . . . . . . . . . . . . . . . .    
630	T . . . . . . . . . . . . . . . .    
631	A . . . . . . . . . . . . . . . .    
632	A . . . . . . . . . . . . . . . .    
633	A . . . . . . . . . . . . . . . .    
634	A . . . . . . . . . . . . . . . .    
635	C . . . . . . . . . . . . . . . .    
636	A . . . . . . . . . . . . . . . .    
637	C . . . . . . . . . . . . . . . .    
638	A . . . . . . . . . . . . . . . .    
639	C . . . . . . . . . . . . . . . .    
640	C . . . . . . . . . . . . . . . .    
641	T . . . . . . . . . . . . . . . .    
642	T . . . . . . . . . . . . . . . .    
643	T . . . . . . . . . . . . . . . .    
644	T . . . . . . . . . . . . . . . .    
645	T . . . . . . . . . . . . . . . .    
646	A . . . . . . . . . . . . . . . .    
647	G . . . . . . . . . . . . . . . .    
648	C . . . . . . . . . . . . . . . .    
649	A . . . . . . . . . . . . . . . .    
650	T . . . . . . . . . . . . . . . .    
651	A . . . . . . . . . . . . . . . .    
652	G . . . . . . . . . . . . . . . .    
653	C . . . . . . . . . . . . . . . .    
654	T . . . . . . . . . . . . . . . .    
655	T . . . . . . . . . . . . . . . .    
656	T . . . . . . . . . . . . . . . .    
657	T . . . . . . . . . . . . . . . .    
658	C . . . . . . . . . . . . . . . .    
659	C . . . . . . . . . . . . . . . .    
660	T . . . . . . . . . . . . . . . .    
661	T . . . . . . . . . . . . . . . .    
662	A . . . . . . . . . . . . . . . .    
663	G . . . . . . . . . . . . . . . .    
664	G . . . . . . . . . . . . . . . .    
665	G . . . . . . . . . . . . . . . .    
666	T . . . . . . . . . . . . . . . .    
667	G . . . . . . . . . . . . . . . .    
668	C . . . . . . . . . . . . . . . .    
669	T . . . . . . . . . . . . . . . .    
670	T . . . . . . . . . . . . . . . .    
671	T . . . . . . . . . . . . . . . .    
672	A . . . . . . . . . . . . . . . .    
673	T . . . . . . . . . . . . . . . .    
674	A . . . . . . . . . . . . . . . .    
675	G . . . . . . . . . . . . . . . .    
676	T . . . . . . . . . . . . . . . .    
677	G . . . . . . . . . . . . . . . .    
678	T . . . . . . . . . . . . . . . .    
679	T . . . . . . . . . . . . . . . .    
680	T . . . . . . . . . . . . . . . .    
681	C . . . . . . . . . . . . . . . .    
682	A . . . . . . . . . . . . . . . .    
683	G . . . . . . . . . . . . . . . .    
684	T . . . . . . . . . . . . . . . .    
685	T . . . . . . . . . . . . . . . .    
686	T . . . . . . . . . . . . . . . .    
687	T . . . . . . . . . . . . . . . .    
688	C . . . . . . . . . . . . . . . .    
689	A . . . . . . . . . . . . . . . .    
690	T . . . . . . . . . . . . . . . .    
691	T . . . . . . . . . . . . . . . .    
692	G . . . . . . . . . . . . . . . .    
693	T . . . . . . . . . . . . . . . .    
694	T . . . . . . . . . . . . . . . .    
695	A . . . . . . . . . . . . . . . .    
696	T . . . . . . . . . . . . . . . .    
697	T . . . . . . . . . . . . . . . .    
698	C . . . . . . . . . . . . . . . .    
699	T . . . . . . . . . . . . . . . .    
700	T . . . . . . . . . . . . . . . .    
701	T . . . . . . . . . . . . . . . .    
702	T . . . . . . . . . . . . . . . .    
703	T . . . . . . . . . . . . . . . .    
704	G . . . . . . . . . . . . . . . .    
705	T . . . . . . . . . . . . . . . .    
706	T . . . . . . . . . . . . . . . .    
707	T . . . . . . . . . . . . . . . .    
708	T . . . . . . . . . . . . . . . .    
709	T . . . . . . . . . . . . . . . .    
710	A . . A . . . . . . . . . . . . .    
711	T . . T . . . . . . . . . . . . .    
712	C . . C . . . . . . . . . . . . .    
713	C . . C . . . . . . . . . . . . .    
714	T . . T . . . . . . . . . . . . .    
715	C . . C . . . . . . . . . . . . .    
716	T . . T . . . . . . . . . . . . .    
717	T . . T . . . . . . . . . . . . .    
718	G . . G . . . . . . . . . . . . .    
719	T . . T . . . . . . . . . . . . .    
720	T . . T . . . . . . . . . . . . .    
721	T . . T . . . . . . . . . . . . .    
722	T . . T . . . . . . . . . . . . .    
723	T . . T . . . . . . . . . . . . .    
724	A . . A . . . . . . . . . . . . .    
725	T . . T . . . . . . . . . . . . .    
726	T . . T . . . . . . . . . . . . .    
727	C . . C . C . . . . . . . . . . .    
728	T . . T . T . . . . . . . . . . .    
729	C . . C . C . . . . . . . . . . .    
730	T . . T . T . . . . . . . . . . .    
731	T . . T . T . . . . . . . . . . .    
732	G . . G . G . . . . . . . . . . .    
733	T . . T . T . . . . . . . . . . .    
734	G . . G . G . . . . . . . . . . .    
735	T . . T . T . . . . . . . . . . .    
736	C . . C . C . . . . . . . . . . .    
737	G . . G . G . . . . . . . . . . .    
738	T . . T . T . . . . . . . . . . .    
739	A . . A . A . . . . . . . . . . .    
740	A . . A . A . . . . . . . . . . .    
741	A . . A . A . . . . . . . . . . .    
742	T . . T . T . . . . . . . . . . .    
743	A . . A . A . . . . . . . . . . .    
744	T . . T . T . . . . . . . . . . .    
745	T . . T . T . . . . . . . . . . .    
746	T . . T . T . . . . . . . . . . .    
747	C . . C . C . . . . . . . . . . .    
748	A . . A . A . . . . . . . . . . .    
749	G . . G . G . . . . . . . . . . .    
750	T . . T . T . . . . . . . . . . .    
751	T . . T . T . . . . . . . . . . .    
752	T . . T . T . . . . . . . . . . .    
753	T . . T . T . . . . . . . . . . .    
754	T . . T . T . . . . . . . . . . .    
755	A . . A . A . . . . . . . . . . .    
756	T . . T . T . . . . . . . . . . .    
757	T . . T . T . . . . . . . . . . .    
758	T . . T . T . . . . . . . . . . .    
759	T . . T . T . . . . . . . . . . .    
760	C . C C . C . . . . . . . . . . .    
761	A . A A . A . . . . . . . . . . .    
762	T . T T . T . . . . . . . . . . .    
763	T . T T . T . . . . . . . . . . .    
764	G . G G . G . . . . . . . . . . .    
765	T . T T . T . . . . . . . . . . .    
766	T . T T . T . . . . . . . . . . .    
767	T . T T . T . . . . . . . . . . .    
768	T . T T . T . . . . . . . . . . .    
769	T . T T . T . . . . . . . . . . .    
770	A . A A . A . . . . . . . . . . .    
771	T . T T . T . . . . . . . . . . .    
772	T . T T . T . . . . . . . . . . .    
773	T . T T . T . . . . . . . . . . .    
774	G . G G . G . . . . . . . . . . .    
775	T . T T . T . . . . . . . . . . .    
776	T . T T . T . . . . . . . . . . .    
777	T . T T . T . . . . . . . . . . .    
778	T . T T . T . . . . . . . . . . .    
779	C . C C . C . . . . . . . . . . .    
780	T . T T . T . . . . . . . . . . .    
781	T . T T . T . . . . . . . . . . .    
782	C . C C . C . . . . . . . . . . .    
783	C . C C . C . . . . . . . . . . .    
784	T . T T . T . . . . . . . . . . .    
785	G . G G . G . . . . . . . . . . .    
786	T . T T . T . . . . . . . . . . .    
787	G . G G . G . . . . . . . . . . .    
788	A . A A . A . . . . . . . . . . .    
789	A . A A . A . . . . . . . . . . .    
790	G . G G . G . . . . . . . . . . .    
791	C . C C . C . . . . . . . . . . .    
792	A . A A . A . . . . . . . . . . .    
793	C . C C . C . . . . . . . . . . .    
794	G . G G . G . . . . . . . . . . .    
795	T . T T . T . . . . . . . . . . .    
796	T . T T . T . . . . . . . . . . .    
797	G . G G . G . . . . . . . . . . .    
798	C . C C . C . . . . . . . . . . .    
799	G . G G . G . . . . . . . . . . .    
800	T . T T . T . . . . . . . . . . .    
801	T . T T . T . . . . . . . . . . .    
802	G . G G . G . . . . . . . . . . .    
803	C . C C . C . . . . . . . . . . .    
804	. . A A . A . . . . . . . . . . .    
805	. . T T . T . . . . . . . . . . .    
806	. . T T . T . . . . . . . . . . .    
807	. . C C . C . . . . . . . . . . .    
808	. . C C . C . . . . . . . . . . .    
809	. . A A . A . . . . . . . . . . .    
810	. . T T . T . . . . . . . . . . .    
811	. . G G . G . . . . . . . . . . .    
812	. . T T . T . . . . . . . . . . .    
813	. . C C . C . . . . . . . . . . .    
814	. . G G . G . . . . . . . . . . .    
815	. . G G . G . . . . . . . . . . .    
816	. . A A . A . . . . . . . . . . .    
817	. . A A . A . . . . . . . . . . .    
818	. . A A . A . . . . . . . . . . .    
819	. . T T . T . . . . . . . . . . .    
820	. . G G . G . . . . . . . . . . .    
821	. . T T . T . . . . . . . . . . .    
822	. . G G . G . . . . . . . . . . .    
823	. . C C . C . . . . . . . . . . .    
824	. . T T . T . . . . . . . . . . .    
825	. . G G . G . . . . . . . . . . .    
826	. . T T . T . . . . . . . . . . .    
827	. . A A . A . . . . . . . . . . .    
828	. . T T . T . . . . . . . . . . .    
829	. . A A . A . . . . . . . . . . .    
830	. . A A . A . . . . . . . . . . .    
831	. . A A . A . . . . . . . . . . .    
832	. . T T . T . . . . . . . . . . .    
833	. . A A . A . . . . . . . . . . .    
834	. . A A . A . . . . . . . . . . .    
835	. . A A . A . . . . . . . . . . .    
836	. . G G . G . . . . . . . . . . .    
837	. . C C . C . . . . . . . . . . .    
838	. . T T . T . . . . . . . . . . .    
839	. . T T . T . . . . . . . . . . .    
840	. . G G . G . . . . . . . . . . .    
841	. . A A . A . . . . . . . . . . .    
842	. . T T . T . . . . . . . . . . .    
843	. . T T . T . . . . . . . . . . .    
844	. . G G . G . . . . . . . . . . .    
845	. . G G . G . . . . . . . . . . .    
846	. . A A . A . . . . . . . . . . .    
847	. . T T . T . . . . . . . . . . .    
848	. . T T . T . . . . . . . . . . .    
849	. . T T . T . . . . . . . . . . .    
850	. . G G . G . . . . . . . . . . .    
851	. . A A . A . . . . . . . . . . .    
852	. . C C . C . . . . . . . . . . .    
853	. . A A . A . . . . . . . . . . .    
854	. . T T . T . . . . . . . . . . .    
855	. . C C . C . . . . . . . . . . .    
856	. . G G . G . . . . . . . . . . .    
857	. . C C . C . . . . . . . . . . .    
858	. . A A . A . . . . . . . . . . .    
859	. . C C . C . . . . . . . . . . .    
860	. . T T . T . . . . . . . . . . .    
861	. . C C . C . . . . . . . . . . .    
862	. . T T . T . . . . . . . . . . .    
863	. . C C . C . . . . . . . . . . .    
864	. . T T . T . . . . . . . . . . .    
865	. . C C . C . . . . . . . . . . .    
866	. . T T . T . . . . . . . . . . .    
867	. . G G . G . . . . . . . . . . .    
868	. . C C . C . . . . . . . . . . .    
869	. . T T . T . . . . . . . . . . .    
870	. . C C . C . . . . . . . . . . .    
871	. . C C . C . . . . . . . . . . .    
872	. . G G . G . . . . . . . . . . .    
873	. . C C . C . . . . . . . . . . .    
874	. . T T . T . . . . . . . . . . .    
875	. . A A . A . . . . . . . . . . .    
876	. . A A . A . . . . . . . . . . .    
877	. . C C . C . . . . . . . . . . .    
878	. . T T T T . . . . . . . . . . .    
879	. . T T T T . . . . . . . . . . .    
880	. . A A A A . . . . . . . . . . .    
881	. . G G G G . . . . . . . . . . .    
882	. . T T T T . . . . . . . . . . .    
883	. . A A A A . . . . . . . . . . .    
884	. . G G G G . . . . . . . . . . .    
885	. . A A A A . . . . . . . . . . .    
886	. . A A A A . . . . . . . . . . .    
887	. . A A A A . . . . . . . . . . .    
888	. . T T T T . . . . . . . . . . .    
889	. . C C C C . . . . . . . . . . .    
890	. . T T T T . . . . . . . . . . .    
891	. . C C C C . . . . . . . . . . .    
892	. . T T T T . . . . . . . . . . .    
893	. . A A A A . . . . . . . . . . .    
894	. . G G G G . . . . . . . . . . .    
895	. . A A A A . . . . . . . . . . .    
896	. . G G G G . . . . . . . . . . .    
897	. . C C C C . . . . . . . . . . .    
898	. . A A A A . . . . . . . . . . .    
899	. . A A A A . . . . . . . . . . .    
900	. . A A A A . . . . . . . . . . .    
901	. . A A A A . . . . . . . . . . .    
902	. . A A A A . . . . . . . . . . .    
903	. . C C C C . . . . . . . . . . .    
904	. . A A A A . . . . . . . . . . .    
905	. . G G G G . . . . . . . . . . .    
906	. . T T T T . . . . . . . . . . .    
907	. . C C C C . . . . . . . . . . .    
908	. . A A A A . . . . . . . . . . .    
909	. . A A A A . . . . . . . . . . .    
910	. . A A A A . . . . . . . . . . .    
911	. . A A A A . . . . . . . . . . .    
912	. . C C C C . . . . . . . . . . .    
913	. . A A A A . . . . . . . . . . .    
914	. . G G G G . . . . . . . . . . .    
915	. . C C C C . . . . . . . . . . .    
916	. . A A A A . . . . . . . . . . .    
917	. . G G G G . . . . . . . . . . .    
918	. C C C C C . . . . . . . . . . .    
919	. A A A A A . . . . . . . . . . .    
920	. A A A A A . . . . . . . . . . .    
921	. C C C C C . . . . . . . . . . .    
922	. T T T T T . . . . . . . . . . .    
923	. A A A A A . . . . . . . . . . .    
924	. T T T T T . . . . . . . . . . .    
925	. T T T T T . . . . . . . . . . .    
926	. A A A A A . . . . . . . . . . .    
927	. A A A A A . . . . . . . . . . .    
928	. A A A - A . . . . . . . . . . .    
929	. T T T T T . . . . . . . . . . .    
930	. T T T T T . . . . . . . . . . .    
931	. C C C C C . . . . . . . . . . .    
932	. A A A A A . . . . . . . . . . .    
933	. A A A A A . . . . . . . . . . .    
934	. A A A A A . . . . . . . . . . .    
935	. T T T T T . . . . . . . . . . .    
936	. G G G G G . . . . . . . . . . .    
937	. A A A A A . . . . . . . . . . .    
938	. A A A A A . . . . . . . . . . .    
939	. A A A A A . . . . . . . . . . .    
940	. A A A A A . . . . . . . . . . .    
941	. C C C C C . . . . . . . . . . .    
942	. A A A A A . . . . . . . . . . .    
943	. A A A A A . . . . . . . . . . .    
944	. C C C C C . . . . . . . . . . .    
945	. A A A A A . . . . . . . . . . .    
946	. A A A A A . . . . . . . . . . .    
947	. C C C C C . . . . . . . . . . .    
948	. T T T T T . . . . . . . . . . .    
949	. T T T T T . . . . . . . . . . .    
950	. C C C C C C . . C C . . . . . .    
951	. A A A A A A . . A A . . . . . .    
952	. T T T T T T . . T T . . . . . .    
953	. A A A A A A . . A A . . . . . .    
954	. C C C C C C . . C C . . . . . .    
955	. A A A A A A . . A A . . . . . .    
956	. A A A A A A . . A A . . . . . .    
957	. A A A A A A . . A A . . . . . .    
958	. T T T T T T . . T T . . . . . .    
959	. A A A A A A . . A A . . . . . .    
960	. C C C C C C . . C C . . . . . .    
961	. A A A A A A . . A A . . . . . .    
962	. T T T T T T . . T T . . . . . .    
963	. T T T T T T . . T T . . . . . .    
964	. T T T T T T . . T T . . . . . .    
965	. C C C C C C . . C C . . . . . .    
966	. T T T T T T . . T T . . . . . .    
967	. T T T T T T . . T T . . . . . .    
968	. A A A A A A . . A A . . . . . .    
969	. G G G G G G . . G G . . . . . .    
970	. T T T T T T . . T T . . . . . .    
971	. G G G G G G . . G G . . . . . .    
972	. C C C C C C . . C C . . . . . .    
973	. A A A A A A A . A A . . . . . .    
974	. T T T T T T T . T T . . . . . .    
975	. A A A A A A A . A A . . . . . .    
976	. T T T T T T T . T T . . . . . .    
977	. T T T T T T T . T T . . . . . .    
978	. T T T T T T T . T T . . . . . .    
979	. T T T T T T T . T T . . . . . .    
980	. C C C C C C C . C C . . . . . .    
981	. A A A A A A A . A A . . . . . .    
982	. T T T T T T T . T T . . . . . .    
983	. A A A A A A A . A A . . . . . .    
984	. T T T T T T T . T T . . . . . .    
985	. C C C C C C C . C C . . . . . .    
986	. A A A A A A A . A A . . . . . .    
987	. A A A A A A A . A A . . . . . .    
988	. A A A A A A A . A A . . . . . .    
989	. C C C C C C C . C C . . . . . .    
990	. A A A A A A A . A A . . . . . .    
991	. T T T T T T T . T T . . . . . .    
992	. G G G G G G G . G G . . . . . .    
993	. A A A A A A A . A A . . . . . .    
994	. T T T T T T T . T T . . . . . .    
995	. C C C C C C C . C C . . . . . .    
996	. A A A A A A A . A A . . . . . .    
997	. T T T T T T T . T T . . . . . .    
998	. G G G G G G G . G G . . . . . .    
999	. C C C C C C C . C C . . . . . .    
1000	. A A A A A A A . A A . . . . . .    
1001	. T T T T T T T . T T . . . . . .    
1002	. T T T T T T T . T T . . . . . .    
1003	. T T T T T T T . T T . . . . . .    
1004	. T T T T T T T . T T . . . . . .    
1005	. T T T T T T T . T T . . . . . .    
1006	. T T T T T T T . T T . . . . . .    
1007	. T T T T T T T . T T . . . . . .    
1008	. T T T T T T T . T T . . . . . .    
1009	. A A A A A A A A A A . . . . . .    
1010	. A A A A A A A A A A . . . . . .    
1011	. A A A A A A A A A A . . . . . .    
1012	. G G G G G G G G G G . . . . . .    
1013	. A A A A A A A A A A . . . . . .    
1014	. C C C C C C C C C C . . . . . .    
1015	. A A A A A A A A A A . . . . . .    
1016	. A A A A A A A A A A . . . . . .    
1017	. T T T T T T T T T T . . . . . .    
1018	. C C C C C C C C C C . . . . . .    
1019	. A A A A A A A A A A . . . . . .    
1020	. T T T T T T T T T T . . . . . .    
1021	. T T T T T T T T T T . . . . . .    
1022	. A A A A A A A A A A . . . . . .    
1023	. T T T T T T T T T T . . . . . .    
1024	. T T T T T T T T T T . . . . . .    
1025	. C C C C C C C C C C . . . . . .    
1026	. C C C C C C C C C C . . . . . .    
1027	. A A A A A A A A A A . . . . . .    
1028	. T T T T T T T T T T . . . . . .    
1029	. G G G G G G G G G G . . . . . .    
1030	. T T T T T T T T T T . . . . . .    
1031	. C C C C C C C C C C . . . . . .    
1032	. A A A A A A A A A A . . . . . .    
1033	. A A A A A A A A A A . . . . . .    
1034	. G G G G G G G G G G . . . . . .    
1035	. T T T T T T T T T T . . . . . .    
1036	. A A A A A A A A A A . . . . . .    
1037	. T T T T T T T T T T . . . . . .    
1038	. T T T T T T T T T T . . . . . .    
1039	. T T T T T T T T T T . . . . . .    
1040	. T T T T T T T T T T . . . . . .    
1041	. T T T T T T T T T T . . . . . .    
1042	. T T T T T T T T T T . . . . . .    
1043	. A A A A A A A A A A . . . . . .    
1044	. T T T T T T T T T T . . . . . .    
1045	. A A A A A A A A A A . . . . . .    
1046	. G G G G G G G G G G . . . . . .    
1047	. C C C C C C C C C C . . . . . .    
1048	. A A A A A A A A A A . . . . . .    
1049	. A A A A A A A A A A . . . . . .    
1050	. A A A A A A A A A A . . . . . .    
1051	. A A A A A A A A A A . . . . . .    
1052	. T T T T T T T T T T . . . . . .    
1053	. T T T T T T T T T T . . . . . .    
1054	. A A A A A A A A A A . . . . . .    
1055	. A A A A A A A A A A . . . . . .    
1056	. A A A A A A A A A A . . . . . .    
1057	. C C C C C C C C C C . . . . . .    
1058	. T T T T T T T T T T . . . . . .    
1059	. A A A A A A A A A A . . . . . .    
1060	. G G G G G G G G G G . . . . . .    
1061	. T T T T T T T T T T . . . . . .    
1062	. G G G G G G G G G G . . . . . .    
1063	. G G G G G G G G G G . . . . . .    
1064	. T T T T T T T T T T . . . . . .    
1065	. A A A A A A A A A A . . . . . .    
1066	. T T T T T T T T T T . . . . . .    
1067	. T T T T T T T T T T . . . . . .    
1068	. A A A A A A A A A A . . . . . .    
1069	. T T T T T T T T T T . . . . . .    
1070	. C C C C C C C C C C . . . . . .    
1071	. A A A A A A A A A A . . . . . .    
1072	. T T T T T T T T T T . . . . . .    
1073	. A A A A A A A A A A . . . . . .    
1074	. G G G G G A G G G G . . . . . .    
1075	. A A A A A A A A A A . . . . . .    
1076	. G G G G G G G G G G . . . . . .    
1077	. C C C C C C C C C C . . . . . .    
1078	. A A A A A A A A A A . . . . . .    
1079	. G G G G G G G G G G . . . . . .    
1080	. T T T T T T T T T T . . . . . .    
1081	. G G G G G G G G G G . . . . . .    
1082	. C C C C C C C C C C . . . . . .    
1083	. T T T T T T T T T T . . . . . .    
1084	. A A A A A A A A A A . . . . . .    
1085	. T T T T T T T T T T . . . . . .    
1086	. G G G G G G G G G G . . . . . .    
1087	. A A A A A A A A A A . . . . . .    
1088	. C C C C C C C C C C . . . . . .    
1089	. C C C C C C C C C C . . . . . .    
1090	. T T T T T T T T T T . . . . . .    
1091	. T T T T T T T T T T . . . . . .    
1092	. G G G G G G G G G G . . . . . .    
1093	. T T T T T T T T T T . . . . . .    
1094	. G G G G G G G G G G . . . . . .    
1095	. G G G G G G G G G G . . . . . .    
1096	. G G G G G G G G G G . . . . . .    
1097	. A A A A A A A A A A . . . . . .    
1098	. T T T T T T T T T T . . . . . .    
1099	. A A A A A A A A A A . . . . . .    
1100	. G G G G G G G G G G . . . . . .    
1101	. T T T T T T T T T T . . . . . .    
1102	. C C C C C C C C C C . . . . . .    
1103	. C C C C C C C C C C . . . . . .    
1104	. C C C C C C C C C C . . . . . .    
1105	. T T T T T T T T T T . . . . . .    
1106	. C C C C C C C C C C . . . . . .    
1107	. G G G G G G G G G G . . . . . .    
1108	. T T T T T T T T T T . . . . . .    
1109	. T T T T T T T T T T . . . . . .    
1110	. C C C C C C C C C C . . . . . .    
1111	. T T T T T T T T T T . . . . . .    
1112	. C C C C C C C C C C . . . . . .    
1113	. A A A A A A A A A A . . . . . .    
1114	. A A A A A A A A A A . . . . . .    
1115	. A A A A A A A A A A . . . . . .    
1116	. T T T T T T T T T T . . . . . .    
1117	. A A A A A A A A A A . . . . . .    
1118	. A A A A A A A A A A . . . . . .    
1119	. T T T T T T T T T T . . . . . .    
1120	. G G G G G G G G G G . . . . . .    
1121	. A A A A A A A A A A . . . . . .    
1122	. A A A A A A A A A A . . . . . .    
1123	. A A A A A A A A A A . . . . . .    
1124	. A A A A A A A A A A . . . . . .    
1125	. A A A A A A A A A A . . . . . .    
1126	. C C C C C C C C C C . . . . . .    
1127	. A A A A A A A A A A . . . . . .    
1128	. C C C C C C C C C C . . . . . .    
1129	. A A A A A A A A A A . . . . . .    
1130	. T T T T T T T T T T . . . . . .    
1131	. G G G G G G G G G G . . . . . .    
1132	. A A A A A A A A A A . . . . . .    
1133	. T T T T T T T T T T . . . . . .    
1134	. C C C C C C C C C C . . . . . .    
1135	. A A A A A A A A A A . A . . . .    
1136	. A A A A A A A A A A . A . . . .    
1137	. T T T T T T T T T T . T . . . .    
1138	. C C C C C C C C C C . C . . . .    
1139	. T T T T T T T T T T . T . . . .    
1140	. T T T T T T T T T T . T . . . .    
1141	. T T T T T T T T T T . T . . . .    
1142	. C C C C C C C C C C . C . C . .    
1143	. C T T T T T T T T T . T . T . .    
1144	. . A A A A A A A A A A A . A . .    
1145	. . T T T T T T T T T T T . T . .    
1146	. . A A A A A A A A A A A . A . .    
1147	. . T T T T T T T T T T T . T . .    
1148	. . C C C C C C C C C C C . C . .    
1149	. . T T T T T T T T T T T . T . .    
1150	. . T T T T T T T T T T T . T . .    
1151	. . C C C C C C C C C C C . C . .    
1152	. . T T T T T T T T T T T . T . .    
1153	. . G G G G G G G G G G G . G . .    
1154	. . T T T T T T T T T T T . T . .    
1155	. . A A A A A A A A A A A . A . .    
1156	. . C C C C C C C C C C C . C . .    
1157	. . T T T T T T T T T T T . T . .    
1158	. . T T T T T T T T T T T . T . .    
1159	. . T T T T T T T T T T T . T . .    
1160	. . T T T T T T T T T T T . T . .    
1161	. . A A A A A A A A A A A . A . .    
1162	. . T T T T T T T T T T T . T . .    
1163	. . A A A A A A A A A A A . A . .    
1164	. . A A A A A A A A A A A . A . .    
1165	. . T T T T T T T T T T T . T . .    
1166	. . G G G G G G G G G G G . G . .    
1167	. . G G G G G G G G G G G . G . .    
1168	. . G G G G G G G G G G G . G . .    
1169	. . A A A A A A A A A A A . A . .    
1170	. . G G G G G G G G G G G . G . .    
1171	. . A A A A A A A A A A A . A . .    
1172	. . A A A A A A A A A A A . A . .    
1173	. . A A A A A A A A A A A . A . .    
1174	. . C C C C C C C C C C C . C C .    
1175	. . A A A A A A A A A A A . A A A    
1176	. . T T T T T T T T T T T . T T T    
1177	. . T T T T T T T T T T T . T T T    
1178	. . A A A A A A A A A A A . A A A    
1179	. . C C C C C C C C C C C . C C C    
1180	. . C C C C C C C C C C C . C C C    
1181	. . C C C C C C C C C C C . C C C    
1182	. . T T T T T T T T T T T . T T T    
1183	. . A A A A A A A A A A A . A A A    
1184	. . T T T T T T T T T T T . T T T    
1185	. . A A A A A A A A A A A . A A A    
1186	. . T T T T T T T T T T T . T T T    
1187	. . C C C C C C C C C C C . C C C    
1188	. . A A A A A A A A A A A . A A A    
1189	. . A A A A A A A A A A A . A A A    
1190	. . A A A A A A A A A A A . A A A    
1191	. . T T T T T T T T T T T . T T T    
1192	. . G G G G G G G G G G G . G G G    
1193	. . A A A A A A A A A A A . A A A    
1194	. . C C C C C C C C C C C . C C C    
1195	. . T T T T T T T T T T T . T T T    
1196	. . T T T T T T T T T T T . T T T    
1197	. . G G G G G G G G G G G . A G G    
1198	. . A A A A A A A A A A A . A A A    
1199	. . T T T T T T T T T T T . T T T    
1200	. . G G G G G G G G G G G . G G G    
1201	. . G G G G G G G G G G G . G G G    
1202	. . A A A A A A A A A A A . A A A    
1203	. . C C C C C C C C C C C . C C C    
1204	. . A A A A A A A A A A A . A A A    
1205	. . C C C C C C C C C C C . C C C    
1206	. . A A A A A A A A A A A . A A A    
1207	. . C C C C C C C C C C C . C C C    
1208	. . A A A A A A A A A A A . A A A    
1209	. . C C C C C C C C C C C . C C C    
1210	. . - - - A - A A - A - A . - A A  7	 indel  
1211	. . - - - C - C C - C - C . - C C  7	 indel  
1212	. . T T T T T T T T T T T . T T T    
1213	. . A A A A A A A A A A A . A A A    
1214	. . T T T T T T T T T T T . T T T    
1215	. . A A A A A A A A A A A . A A A    
1216	. . C C C C C C C C C C C . C C C    
1217	. . C C C C C C C C C C C . C C C    
1218	. . C C C C C C C C C C C . C C C    
1219	. . A A A A A A A A A A A . A A A    
1220	. . C C C C C C C C C C C . C C C    
1221	. . A A A A A A A A A A A . A A A    
1222	. . T T T T T T T T T T T . T T T    
1223	. . T T T T T T T T T T T . T T T    
1224	. . C C C C C C C C C C C . C C C    
1225	. . C C A C C C C C C A C . C C C    
1226	. . C C C C C C C C C C C . C C C    
1227	. . G G G G G G G G G G G . G G G    
1228	. . A A A A A A A A A A A . A A A    
1229	. . A A A A A A A A A A A . A A A    
1230	. . A A A A A A A A A A A . A A A    
1231	. . C C C C C C C C C C C . C C C    
1232	. . A A A A A A A A A A A . A A A    
1233	. . A A A A A A A A A A A . A A A    
1234	. . A A A A A A A A A A A . A A A    
1235	. . C C C C C C C C C C C . C C C    
1236	. . C C C C C C C C C C C . C C C    
1237	. . C C C C C C C C C C C . C C C    
1238	. . C C C C C C C C C C C . C C C    
1239	. . C C C C C C C C C C C . C C C    
1240	. . T T T T T T T T T T T . T T T    
1241	. . G G G G G G G G G G G . G G G    
1242	. . A A A A A A A A A A A . A A A    
1243	. . T T T T T T T T T T T . T T T    
1244	. . C C C C C C C C C C C . C C C    
1245	. . G G G G G G G G G G G . G G G    
1246	. . A A A A A A A A A A A . A A A    
1247	. . G G G G G G G G G G G . G G G    
1248	. . A A A A A A A A A A A . A A A    
1249	. . A A A A A A A A A A A . A A A    
1250	. . G G G G G G G G G G G . G G G    
1251	. . T T T T T T T T T T T . T T T    
1252	. . A A A A A A A A A A A . A A A    
1253	. . C C C C C C C C C C C . C C C    
1254	. . T T T T T T T T T T T . T T T    
1255	. . G G G G G G G G G G G . G G G    
1256	. . A A A A A A A A A A A . A A A    
1257	. . G G G G G G G G G G G . G G G    
1258	. . A A A A A A A A A A A . A A A    
1259	. . T T T T T T T T T T T . T T T    
1260	. . G G G G G G G G G G G . G G G    
1261	. . A A A A A A A A A A A . A A A    
1262	. . A A A A A A A A A A A . A A A    
1263	. . G G G G A G G G G G A . G G G    
1264	. . A A A A A A A A A A A . A A A    
1265	. . A G G G G G G G G G G . G G G    
1266	. . . A A A A A A A A A A A A A A    
1267	. . . A A A A A A A A A A A A A A    
1268	. . . G G G G G G G G G G G G G G    
1269	. . . G G G G G G G G G G G G G G    
1270	. . . A A A A A A A A A A A A A A    
1271	. . . G G G G G G G G G G G G G G    
1272	. . . G G G G G G G G G G G G G G    
1273	. . . G G G G G G G G G G G G G G    
1274	. . . T T T T T T T T T T T T T T    
1275	. . . A A A A A A A A A A A A A A    
1276	. . . G G G G G G G G G G G G G G    
1277	. . . G G G G G G G G G G G G G G    
1278	. . . A A A A A A A A A A A A A A    
1279	. . . A A A A A A A A A A A A A A    
1280	. . . T T T T T T T T T T T T T T    
1281	. . . G G G G G G G G G G G G G G    
1282	. . . G G G G G G G G G G G G G G    
1283	. . . C C C C C C C C C C C C C C    
1284	. . . A A A A A A A A A A A A A A    
1285	. . . T T T T T T T T T T T T T T    
1286	. . . G G G G G G G G G G G G G G    
1287	. . . C C C C C C C C C C C C C C    
1288	. . . C C C C C C C C C C C C C C    
1289	. . . T T T T T T T T T T T T T T    
1290	. . . G G G G G G G G G G G G G G    
1291	. . . T T T T T T T T T T T T T T    
1292	. . . A A A A A A A A A A A A A A    
1293	. . . T T T T T T T T T T T T T T    
1294	. . . C C C C C C C C C C C C C C    
1295	. . . C C C C C C C C C C C C C C    
1296	. . . A A A A A A A A A A A A A A    
1297	. . . T T T T T T T T T T T T T T    
1298	. . . G G G G G G G G G G G G G G    
1299	. . . G G G G G G G G G G G G G G    
1300	. . . A A A A A A A A A A A A A A    
1301	. . . G G G G G G G G G G G G G G    
1302	. . . A A A A A A A A A A A A A A    
1303	. . . T T T T T T T T T T T T T T    
1304	. . . C C C C C C C C C C C C C C    
1305	. . . A A A A A A A A A A A A A A    
1306	. . . G G G G G G G G G G G G G G    
1307	. . . C C C C C C C C C C C C C C    
1308	. . . T T T T T T T T T T T T T T    
1309	. . . C C C C C C C C C C C C C C    
1310	. . . T T T T T T T T T T T T T T    
1311	. . . A A A A A A A A A A A A A A    
1312	. . . A A A A A A A A A A A A A A    
1313	. . . C C C C C C C C C C C C C C    
1314	. . . C C C C C C C C C C C C C C    
1315	. . . A A A A A A A A A A A A A A    
1316	. . . C C C C C C C C C C C C C C    
1317	. . . T T T T T T T T T T T T T T    
1318	. . . C C C C C C C C C C C C C C    
1319	. . . T T T T T T T T T T T T T T    
1320	. . . G G G G G G G G G G G G G G    
1321	. . . G G G G G G G G G G G G G G    
1322	. . . C C C C C C C C C C C C C C    
1323	. . . A A A A A A A A A A A A A A    
1324	. . . T T T T T T T T T T T T T T    
1325	. . . G G G G G G G G G G G G G G    
1326	. . . T T T T T T T T T T T T T T    
1327	. . . T T T T T T T T T T T T T T    
1328	. . . C C C C C C C C C C C C C C    
1329	. . . T T T T T T T T T T T T T T    
1330	. . . T T T T T T T T T T T T T T    
1331	. . . T T T T T T T T T T T T T T    
1332	. . . C C C C C C C C C C C C C C    
1333	. . . A A A A A A A A A A A A A A    
1334	. . . G G G G G G G G G G G G G G    
1335	. . . T T T T T T T T T T T T T T    
1336	. . . A A A A A A A A A A A A A A    
1337	. . . G G G G G G G G G G G G G G    
1338	. . . G G G G G G G G G G G G G G    
1339	. . . G G G G G G G G G G G G G G    
1340	. . . C C C C C C C C C C C C C C    
1341	. . . T T T T T T T T T T T T T T    
1342	. . . T T T T T T T T T T T T T T    
1343	. . . T T T T T T T T T T T T T T    
1344	. . . C C C C C C C C C C C C C C    
1345	. . . T T T T T T T T T T T T T T    
1346	. . . C C C C C C C C C C C C C C    
1347	. . . T T T T T T T T T T T T T T    
1348	. . . T T T T T T T T T T T T T T    
1349	. . . G G G G G G G G G G G G G G    
1350	. . . T T T T T T T T T T T T T T    
1351	. . . T T T T T T T T T T T T T T    
1352	. . . T T T T T T T T T T T T T T    
1353	. . . G G G G G G G G G G G G G G    
1354	. . . C C C C C C C C C C C C C C    
1355	. . . T T T T T T T T T T T T T T    
1356	. . . T T T T T T T T T T T T T T    
1357	. . . G T T T T T T T T T T T T T    
1358	. . . . . T T T T T T T T T T T T    
1359	. . . . . C C C C C C C C C C C C    
1360	. . . . . T T T T T T T T T T T T    
1361	. . . . . T T T T T T T T T T T T    
1362	. . . . . G G G G G G G G G G G G    
1363	. . . . . C C C C C C C C C C C C    
1364	. . . . . T T T T T T T T T T T T    
1365	. . . . . G G G G G G G G G G G G    
1366	. . . . . A A A A A A A A A A A A    
1367	. . . . . G G G G G G G G G G G G    
1368	. . . . . C C C C C C C C C C C C    
1369	. . . . . T T T T T T T T T T T T    
1370	. . . . . C C C C C C C C C C C C    
1371	. . . . . A A A A A A A A A A A A    
1372	. . . . . G G G G G G G G G G G G    
1373	. . . . . G G G G G G G G G G G G    
1374	. . . . . C C C C C C C C C C C C    
1375	. . . . . G G G G G G G G G G G G    
1376	. . . . . . G G G G G G G G G G G    
1377	. . . . . . G G G G G G G G G G G    
1378	. . . . . . G G G G G G G G G G G    
1379	. . . . . . T T T T T T T T T T T    
1380	. . . . . . A A A A A A A A A A A    
1381	. . . . . . G G G G G G G G G G G    
1382	. . . . . . C C C C C C C C C C C    
1383	. . . . . . A A A A A A A A A A A    
1384	. . . . . . T T T T T T T T T T T    
1385	. . . . . . C C C C C C C C C C C    
1386	. . . . . . A A A A A A A A A A A    
1387	. . . . . . C C C C C C C C C C C    
1388	. . . . . . A A A A A A A A A A A    
1389	. . . . . . C C C C C C C C C C C    
1390	. . . . . . A A A A A A A A A A A    
1391	. . . . . . C C C C C C C C C C C    
1392	. . . . . . A A A A A A A A A A A    
1393	. . . . . . A A A A A A A A A A A    
1394	. . . . . . A A A A A A A A A A A    
1395	. . . . . . G G G G G G G G G G G    
1396	. . . . . . T T T T T T T T T T T    
1397	. . . . . . G G G G G G G G G G G    
1398	. . . . . . G G G G G G G G G G G    
1399	. . . . . . C C C C C C C C C C C    
1400	. . . . . . C C C C C C C C C C C    
1401	. . . . . . A A A A A A A A A A A    
1402	. . . . . . C C C C C C C C C C C    
1403	. . . . . . C C C C C C C C C C C    
1404	. . . . . . A A A A A A A A A A A    
1405	. . . . . . T T T T T T T T T T T    
1406	. . . . . . G G G G G G G G G G G    
1407	. . . . . . A A A A A A A A A A A    
1408	. . . . . . C C C C C C C C C C C    
1409	. . . . . . A A A A A A A A A A A    
1410	. . . . . . A A A A A A A A A A A    
1411	. . . . . . G G G G G G G G G G G    
1412	. . . . . . G G G G G G G G G G G    
1413	. . . . . . A A A A A A A A A A A    
1414	. . . . . . T T T T T T T T T T T    
1415	. . . . . . G G G G G G G G G G G    
1416	. . . . . . A A A A A A A A A A A    
1417	. . . . . . A A A A A A A A A A A    
1418	. . . . . . T T T T T T T T T T T    
1419	. . . . . . C C C C C C C C C C C    
1420	. . . . . . C C C C C C C C C C C    
1421	. . . . . . A A A A A A A A A A A    
1422	. . . . . . C C C C C C C C C C C    
1423	. . . . . . A A A A A A A A A A A    
1424	. . . . . . G G G G G G G G G G G    
1425	. . . . . . C C C C C C C C C C C    
1426	. . . . . . C C C C C C C C C C C    
1427	. . . . . . A A A A A A A A A A A    
1428	. . . . . . A A A A A A A A A A A    
1429	. . . . . . A A A A A A A A A A A    
1430	. . . . . . G G G G G G G G G G G    
1431	. . . . . . A A A A A A A A A A A    
1432	. . . . . . A A A A A A A A A A A    
1433	. . . . . . C C C C C C C C C C C    
1434	. . . . . . A A A A A A A A A A A    
1435	. . . . . . A A A A A A A A A A A    
1436	. . . . . . C C C C C C C C C C C    
1437	. . . . . . C C C C C C C C C C C    
1438	. . . . . . A A A A A A A A A A A    
1439	. . . . . . C C C C C C C C C C C    
1440	. . . . . . A A A A A A A A A A A    
1441	. . . . . . G G G G G G G G G G G    
1442	. . . . . . G G G G G G G G G G G    
1443	. . . . . . A A A A A A A A A A A    
1444	. . . . . . A A A A A A A A A A A    
1445	. . . . . . G G G G G G G G G G G    
1446	. . . . . . T T T T T T T T T T T    
1447	. . . . . . T T T T T T T T T T T    
1448	. . . . . . C C C C C C C C C C C    
1449	. . . . . . C C C C C C C C C C C    
1450	. . . . . . A A A A A A A A A A A    
1451	. . . . . . A A A A A A A A A A A    
1452	. . . . . . C C C C C C C C C C C    
1453	. . . . . . T T T T T T T T T T T    
1454	. . . . . . G G G G G G G G G G G    
1455	. . . . . . T T T T T T T T T T T    
1456	. . . . . . C C C C C C C C C C C    
1457	. . . . . . A A A A A A A A A A A    
1458	. . . . . . A A A A A A A A A A A    
1459	. . . . . . C C C C C C C C C C C    
1460	. . . . . . C C C C C C C C C C C    
1461	. . . . . . T T T T T T T T T T T    
1462	. . . . . . C C C C C C C C C C C    
1463	. . . . . . T T T T T T T T T T T    
1464	. . . . . . G G G G G G G G G G G    
1465	. . . . . . T T T T T T T T T T T    
1466	. . . . . . G G G G G G G G G G G    
1467	. . . . . . A A A A A A A A A A A    
1468	. . . . . . C C C C C C C C C C C    
1469	. . . . . . G G G G G G G G G G G    
1470	. . . . . . A A A A A A A A A A A    
1471	. . . . . . T T T T T T T T T T T    
1472	. . . . . . G G G G G G G G G G G    
1473	. . . . . . A A A A A A A A A A A    
1474	. . . . . . G G G G G G G G G G G    
1475	. . . . . . G G G G G G G G G G G    
1476	. . . . . . G G G G G G G G G G G    
1477	. . . . . . G T T T T T T T T T T    
1478	. . . . . . . G G G G G G G G G G    
1479	. . . . . . . A A A A A A A A A A    
1480	. . . . . . . A A A A A A A A A A    
1481	. . . . . . . G G G G G G G G G G    
1482	. . . . . . . C C C C C C C C C C    
1483	. . . . . . . T T T T T T T T T T    
1484	. . . . . . . C C C C C C C C C C    
1485	. . . . . . . T T T T T T T T T T    
1486	. . . . . . . T T T T T T T T T T    
1487	. . . . . . . G G G G G G G G G G    
1488	. . . . . . . C C C C C C C C C C    
1489	. . . . . . . G G G G G G G G G G    
1490	. . . . . . . G G G G G G G G G G    
1491	. . . . . . . A A A A A A A A A A    
1492	. . . . . . . T T T T T T T T T T    
1493	. . . . . . . G G G G G G G G G G    
1494	. . . . . . . G G G G G G G G G G    
1495	. . . . . . . G G G G G G G G G G    
1496	. . . . . . . C C C C C C C C C C    
1497	. . . . . . . A A A A A A A A A A    
1498	. . . . . . . T T T T T T T T T T    
1499	. . . . . . . G G G G G G G G G G    
1500	. . . . . . . C C C C C C C C C C    
1501	. . . . . . . G G G G G G G G G G    
1502	. . . . . . . C C C C C C C C C C    
1503	. . . . . . . A A A A A A A A A A    
1504	. . . . . . . G G G G G G G G G G    
1505	. . . . . . . G G G G G G G G G G    
1506	. . . . . . . G G G G G G G G G G    
1507	. . . . . . . A A A A A A A A A A    
1508	. . . . . . . G G G G G G G G G G    
1509	. . . . . . . C C C C C C C C C C    
1510	. . . . . . . T T T T T T T T T T    
1511	. . . . . . . G G G G G G G G G G    
1512	. . . . . . . T T T T T T T T T T    
1513	. . . . . . . G G G G G G G G G G    
1514	. . . . . . . A A A A A A A A A A    
1515	. . . . . . . G G G G G G G G G G    
1516	. . . . . . . A A A A A A A A A A    
1517	. . . . . . . C C C C C C C C C C    
1518	. . . . . . . A A A A A A A A A A    
1519	. . . . . . . C C C C C C C C C C    
1520	. . . . . . . C C C C C C C C C C    
1521	. . . . . . . C C C C C C C C C C    
1522	. . . . . . . T T T T T T T T T T    
1523	. . . . . . . G G G G G G G G G G    
1524	. . . . . . . C C C C C C C C C C    
1525	. . . . . . . A A A A A A A A A A    
1526	. . . . . . . G G G G G G A G G G    
1527	. . . . . . . A A A A A A A A A A    
1528	. . . . . . . A A A A A A A A A A    
1529	. . . . . . . G G G G G G G G G G    
1530	. . . . . . . T T T T T T T T T T    
1531	. . . . . . . A A A A A A A A A A    
1532	. . . . . . . C C C C C C C C C C    
1533	. . . . . . . T T T T T T T T T T    
1534	. . . . . . . T T T T T T T T T T    
1535	. . . . . . . G G G G G G G G G G    
1536	. . . . . . . G G G G G G G G G G    
1537	. . . . . . . A A A A A A A A A A    
1538	. . . . . . . G G G G G G G G G G    
1539	. . . . . . . C C C C C C C C C C    
1540	. . . . . . . C C C C C C C C C C    
1541	. . . . . . . C C C C C C C C C C    
1542	. . . . . . . G G G G G G G G G G    
1543	. . . . . . . A A A A A A A A A A    
1544	. . . . . . . G G G G G G G G G G    
1545	. . . . . . . T T T T T T T T T T    
1546	. . . . . . . C C C C C C C C C C    
1547	. . . . . . . G G G G G G G G G G    
1548	. . . . . . . T T T T T T T T T T    
1549	. . . . . . . G G G G G G G G G G    
1550	. . . . . . . C C C C C C C C C C    
1551	. . . . . . . A A A A A A A A A A    
1552	. . . . . . . G G G G G G G G G G    
1553	. . . . . . . G G G G G G G G G G    
1554	. . . . . . . G G G G G G G G G G    
1555	. . . . . . . A A A A A A A A A A    
1556	. . . . . . . A A A A A A A A A A    
1557	. . . . . . . G G G G G G G G G G    
1558	. . . . . . . C C C C C C C C C C    
1559	. . . . . . . G G G G G G G G G G    
1560	. . . . . . . T T T T T T T T T T    
1561	. . . . . . . G G G G G G G G G G    
1562	. . . . . . . G G G G G G G G G G    
1563	. . . . . . . A A A A A A A A A A    
1564	. . . . . . . G G G G G G G G G G    
1565	. . . . . . . C C C C C C C C C C    
1566	. . . . . . . A A A A A A A A A A    
1567	. . . . . . . . G G G G G G G G G    
1568	. . . . . . . . G G G G G G G G G    
1569	. . . . . . . . A A A A A A A A A    
1570	. . . . . . . . A A A A A A A A A    
1571	. . . . . . . . G G G G G A G G G    
1572	. . . . . . . . A A A A A A A A A    
1573	. . . . . . . . A A A A A A A A A    
1574	. . . . . . . . G G G G G G G G G    
1575	. . . . . . . . G G G G G G G G G    
1576	. . . . . . . . C C C C C C C C C    
1577	. . . . . . . . C C C C C C C C C    
1578	. . . . . . . . G G G G G G G G G    
1579	. . . . . . . . A A A A A A A A A    
1580	. . . . . . . . C C C C C C C C C    
1581	. . . . . . . . A A A A A A A A A    
1582	. . . . . . . . G G G G G G G G G    
1583	. . . . . . . . A A A A A A A A A    
1584	. . . . . . . . G G G G G A G G G    
1585	. . . . . . . . A A A A A A A A A    
1586	. . . . . . . . G G G G G G G G G    
1587	. . . . . . . . T T T T T T T T T    
1588	. . . . . . . . A A A A A A A A A    
1589	. . . . . . . . G G G G G G G G G    
1590	. . . . . . . . G G G G G G G G G    
1591	. . . . . . . . T T T T T T T T T    
1592	. . . . . . . . C C C C C C C C C    
1593	. . . . . . . . C C C C C C C C C    
1594	. . . . . . . . C C C C C C C C C    
1595	. . . . . . . . G G G G G G G G G    
1596	. . . . . . . . T T T T T T T T T    
1597	. . . . . . . . C C C C C C C C C    
1598	. . . . . . . . C C C C C C C C C    
1599	. . . . . . . . T T T T T T T T T    
1600	. . . . . . . . G G G G G G G G G    
1601	. . . . . . . . G G G G G G G G G    
1602	. . . . . . . . T T T T T T T T T    
1603	. . . . . . . . G G G G G G G G G    
1604	. . . . . . . . G G G G G G G G G    
1605	. . . . . . . . T T T T T T T T T    
1606	. . . . . . . . G G G G G G G G G    
1607	. . . . . . . . G G G G G G G G G    
1608	. . . . . . . . C C C C C C C C C    
1609	. . . . . . . . G G G G G G G G G    
1610	. . . . . . . . G G G G G G G G G    
1611	. . . . . . . . T T T T T T T T T    
1612	. . . . . . . . G G G G G G G G G    
1613	. . . . . . . . G G G G G G G G G    
1614	. . . . . . . . C C C C C C C C C    
1615	. . . . . . . . T T T T T T T T T    
1616	. . . . . . . . G G G G G G G G G    
1617	. . . . . . . . . G G G G G G G G    
1618	. . . . . . . . . A A A A A A A A    
1619	. . . . . . . . . G G G G G G G G    
1620	. . . . . . . . . T T T T T T T T    
1621	. . . . . . . . . A A A A A A A A    
1622	. . . . . . . . . C C C C C C C C    
1623	. . . . . . . . . A A A A A A A A    
1624	. . . . . . . . . G G G G G G G G    
1625	. . . . . . . . . G G G G G G G G    
1626	. . . . . . . . . A A A A A A A A    
1627	. . . . . . . . . C C C C C C C C    
1628	. . . . . . . . . A A A A A A A A    
1629	. . . . . . . . . G G G G G G G G    
1630	. . . . . . . . . T T T T T T T T    
1631	. . . . . . . . . G G G G G G G G    
1632	. . . . . . . . . T G T T T T T T    
1633	. . . . . . . . . C . C C C C C C    
1634	. . . . . . . . . C . C C C C C C    
1635	. . . . . . . . . A . A A A A A A    
1636	. . . . . . . . . G . G G G G G G    
1637	. . . . . . . . . C . C C C C C C    
1638	. . . . . . . . . T . T T T T T T    
1639	. . . . . . . . . T . T T T T T T    
1640	. . . . . . . . . C . C C C C C C    
1641	. . . . . . . . . T . T T T T T T    
1642	. . . . . . . . . C . C C C C C C    
1643	. . . . . . . . . T . T T T T T T    
1644	. . . . . . . . . G . G G G G G G    
1645	. . . . . . . . . G . G G G G G G    
1646	. . . . . . . . . T . T T T T T T    
1647	. . . . . . . . . A . A A A A A A    
1648	. . . . . . . . . G . G G G G G G    
1649	. . . . . . . . . G . G G G G G G    
1650	. . . . . . . . . A . A A A A A A    
1651	. . . . . . . . . G . G G G G G G    
1652	. . . . . . . . . G . G G G G G G    
1653	. . . . . . . . . C . C C C C C C    
1654	. . . . . . . . . C . C C C C C C    
1655	. . . . . . . . . T . T T T T T T    
1656	. . . . . . . . . C . T C C C C C    
1657	. . . . . . . . . C . . C C C C C    
1658	. . . . . . . . . A . . A A A A A    
1659	. . . . . . . . . C . . C C C C C    
1660	. . . . . . . . . C . . C C C C C    
1661	. . . . . . . . . T . . T T T T T    
1662	. . . . . . . . . C . . C C C C C    
1663	. . . . . . . . . C . . C C C C C    
1664	. . . . . . . . . A . . A A A A A    
1665	. . . . . . . . . C . . C C C C C    
1666	. . . . . . . . . C . . C C C C C    
1667	. . . . . . . . . A . . A A A A A    
1668	. . . . . . . . . C . . C C C C C    
1669	. . . . . . . . . T . . T T T T T    
1670	. . . . . . . . . G . . G G G G G    
1671	. . . . . . . . . G . . G G G G G    
1672	. . . . . . . . . G . . G G G G G    
1673	. . . . . . . . . C . . C C C C C    
1674	. . . . . . . . . . . . T T T T T    
1675	. . . . . . . . . . . . C C C C C    
1676	. . . . . . . . . . . . C C C C C    
1677	. . . . . . . . . . . . C C C C C    
1678	. . . . . . . . . . . . T T T T T    
1679	. . . . . . . . . . . . G G G G G    
1680	. . . . . . . . . . . . A A A A A    
1681	. . . . . . . . . . . . A A A A A    
1682	. . . . . . . . . . . . C C C C C    
1683	. . . . . . . . . . . . C C C C C    
1684	. . . . . . . . . . . . A A A A A    
1685	. . . . . . . . . . . . C C C C C    
1686	. . . . . . . . . . . . T T T T T    
1687	. . . . . . . . . . . . C C C C C    
1688	. . . . . . . . . . . . C C C C C    
1689	. . . . . . . . . . . . A A A A A    
1690	. . . . . . . . . . . . T T T T T    
1691	. . . . . . . . . . . . C C C C C    
1692	. . . . . . . . . . . . T T T T T    
1693	. . . . . . . . . . . . C C C C C    
1694	. . . . . . . . . . . . C C C C C    
1695	. . . . . . . . . . . . A A A A A    
1696	. . . . . . . . . . . . C C C C C    
1697	. . . . . . . . . . . . G G G G G    
1698	. . . . . . . . . . . . T T T T T    
1699	. . . . . . . . . . . . C C C C C    
1700	. . . . . . . . . . . . C C C C C    
1701	. . . . . . . . . . . . A A A A A    
1702	. . . . . . . . . . . . G G G G G    
1703	. . . . . . . . . . . . G G G G G    
1704	. . . . . . . . . . . . G G G G G    
1705	. . . . . . . . . . . . G G G G G    
1706	. . . . . . . . . . . . G G G G G    
1707	. . . . . . . . . . . . T T T T T    
1708	. . . . . . . . . . . . A A A A A    
1709	. . . . . . . . . . . . G G G G G    
1710	. . . . . . . . . . . . T T T T T    
1711	. . . . . . . . . . . . A A A A A    
1712	. . . . . . . . . . . . G G G G G    
1713	. . . . . . . . . . . . C C C C C    
1714	. . . . . . . . . . . . C C C C C    
1715	. . . . . . . . . . . . C C C C C    
1716	. . . . . . . . . . . . A A A A A    
1717	. . . . . . . . . . . . C C C C C    
1718	. . . . . . . . . . . . T T T T T    
1719	. . . . . . . . . . . . G G G G G    
1720	. . . . . . . . . . . . C C C C C    
1721	. . . . . . . . . . . . C C C C C    
1722	. . . . . . . . . . . . T T T T T    
1723	. . . . . . . . . . . . C C C C C    
1724	. . . . . . . . . . . . A A A A A    
1725	. . . . . . . . . . . . C C C C C    
1726	. . . . . . . . . . . . A A A A A    
1727	. . . . . . . . . . . . C C C C C    
1728	. . . . . . . . . . . . A A A A A    
1729	. . . . . . . . . . . . C C C C C    
1730	. . . . . . . . . . . . C C C C C    
1731	. . . . . . . . . . . . A A A A A    
1732	. . . . . . . . . . . . T T T T T    
1733	. . . . . . . . . . . . C C C C C    
1734	. . . . . . . . . . . . T T T T T    
1735	. . . . . . . . . . . . T T T T T    
1736	. . . . . . . . . . . . C C C C C    
1737	. . . . . . . . . . . . T T T T T    
1738	. . . . . . . . . . . . G G G G G    
1739	. . . . . . . . . . . . C C C C C    
1740	. . . . . . . . . . . . C C C C C    
1741	. . . . . . . . . . . . T T T T T    
1742	. . . . . . . . . . . . C T C C C    
1743	. . . . . . . . . . . . T . T T T    
1744	. . . . . . . . . . . . C . C C C    
1745	. . . . . . . . . . . . C . C C C    
1746	. . . . . . . . . . . . G . G G G    
1747	. . . . . . . . . . . . T . T T T    
1748	. . . . . . . . . . . . G . G G G    
1749	. . . . . . . . . . . . T . T T T    
1750	. . . . . . . . . . . . C . C C C    
1751	. . . . . . . . . . . . C . C C C    
1752	. . . . . . . . . . . . A . A A A    
1753	. . . . . . . . . . . . C . C C C    
1754	. . . . . . . . . . . . C . C C C    
1755	. . . . . . . . . . . . A . A A A    
1756	. . . . . . . . . . . . A . A A A    
1757	. . . . . . . . . . . . . . G G G    
1758	. . . . . . . . . . . . . . G G G    
1759	. . . . . . . . . . . . . . A A A    
1760	. . . . . . . . . . . . . . G G G    
1761	. . . . . . . . . . . . . . A A A    
1762	. . . . . . . . . . . . . . T T T    
1763	. . . . . . . . . . . . . . G G G    
1764	. . . . . . . . . . . . . . A A A    
1765	. . . . . . . . . . . . . . C C C    
1766	. . . . . . . . . . . . . . A A A    
1767	. . . . . . . . . . . . . . G G G    
1768	. . . . . . . . . . . . . . A A A    
1769	. . . . . . . . . . . . . . G G G    
1770	. . . . . . . . . . . . . . T T T    
1771	. . . . . . . . . . . . . . C C C    
1772	. . . . . . . . . . . . . . T T T    
1773	. . . . . . . . . . . . . . A A A    
1774	. . . . . . . . . . . . . . C C C    
1775	. . . . . . . . . . . . . . G G G    
1776	. . . . . . . . . . . . . . T T T    
1777	. . . . . . . . . . . . . . T T T    
1778	. . . . . . . . . . . . . . C C C    
1779	. . . . . . . . . . . . . . A A A    
1780	. . . . . . . . . . . . . . G G G    
1781	. . . . . . . . . . . . . . G G G    
1782	. . . . . . . . . . . . . . G G G    
1783	. . . . . . . . . . . . . . A A A    
1784	. . . . . . . . . . . . . . C C C    
1785	. . . . . . . . . . . . . . A A A    
1786	. . . . . . . . . . . . . . C C C    
1787	. . . . . . . . . . . . . . G G G    
1788	. . . . . . . . . . . . . . C C C    
1789	. . . . . . . . . . . . . . G G G    
1790	. . . . . . . . . . . . . . A A A    
1791	. . . . . . . . . . . . . . G G G    
1792	. . . . . . . . . . . . . . G G G    
1793	. . . . . . . . . . . . . . A A A    
1794	. . . . . . . . . . . . . . G G G    
1795	. . . . . . . . . . . . . . G G G    
1796	. . . . . . . . . . . . . . C C C    
1797	. . . . . . . . . . . . . . T T T    
1798	. . . . . . . . . . . . . . C C C    
1799	. . . . . . . . . . . . . . C C C    
1800	. . . . . . . . . . . . . . A A A    
1801	. . . . . . . . . . . . . . T T T    
1802	. . . . . . . . . . . . . . G G G    
1803	. . . . . . . . . . . . . . A A A    
1804	. . . . . . . . . . . . . . T T T    
1805	. . . . . . . . . . . . . . G G G    
1806	. . . . . . . . . . . . . . T T T    
1807	. . . . . . . . . . . . . . G G G    
1808	. . . . . . . . . . . . . . C C C    
1809	. . . . . . . . . . . . . . A A A    
1810	. . . . . . . . . . . . . . G G G    
1811	. . . . . . . . . . . . . . A A A    
1812	. . . . . . . . . . . . . . G G G    
1813	. . . . . . . . . . . . . . C C C    
1814	. . . . . . . . . . . . . . G G G    
1815	. . . . . . . . . . . . . . . A A    
1816	. . . . . . . . . . . . . . . T T    
1817	. . . . . . . . . . . . . . . T T    
1818	. . . . . . . . . . . . . . . T T    
1819	. . . . . . . . . . . . . . . C C    
1820	. . . . . . . . . . . . . . . A A    
1821	. . . . . . . . . . . . . . . T T    
1822	. . . . . . . . . . . . . . . G G    
1823	. . . . . . . . . . . . . . . G G    
1824	. . . . . . . . . . . . . . . C C    
1825	. . . . . . . . . . . . . . . T T    
1826	. . . . . . . . . . . . . . . G G    
1827	. . . . . . . . . . . . . . . C C    
1828	. . . . . . . . . . . . . . . C C    
1829	. . . . . . . . . . . . . . . A A    
1830	. . . . . . . . . . . . . . . A A    
1831	. . . . . . . . . . . . . . . A A    
1832	. . . . . . . . . . . . . . . C C    
1833	. . . . . . . . . . . . . . . A A    
1834	. . . . . . . . . . . . . . . G G    
1835	. . . . . . . . . . . . . . . C C    
1836	. . . . . . . . . . . . . . . G G    
1837	. . . . . . . . . . . . . . . G G    
1838	. . . . . . . . . . . . . . . A A    
1839	. . . . . . . . . . . . . . . G G    
1840	. . . . . . . . . . . . . . . G G    
1841	. . . . . . . . . . . . . . . C C    
1842	. . . . . . . . . . . . . . . A A    
1843	. . . . . . . . . . . . . . . C C    
1844	. . . . . . . . . . . . . . . C C    
1845	. . . . . . . . . . . . . . . G G    
1846	. . . . . . . . . . . . . . . A A    
1847	. . . . . . . . . . . . . . . G G    
1848	. . . . . . . . . . . . . . . A A    
1849	. . . . . . . . . . . . . . . C C    
1850	. . . . . . . . . . . . . . . A A    
1851	. . . . . . . . . . . . . . . A A    
1852	. . . . . . . . . . . . . . . G G    
1853	. . . . . . . . . . . . . . . G G    
1854	. . . . . . . . . . . . . . . C C    
1855	. . . . . . . . . . . . . . . A A    
1856	. . . . . . . . . . . . . . . G G    
1857	. . . . . . . . . . . . . . . A A    
1858	. . . . . . . . . . . . . . . C C    
1859	. . . . . . . . . . . . . . . A A    
1860	. . . . . . . . . . . . . . . T T    
1861	. . . . . . . . . . . . . . . A A    
1862	. . . . . . . . . . . . . . . C C    
1863	. . . . . . . . . . . . . . . G G    
1864	. . . . . . . . . . . . . . . T T    
1865	. . . . . . . . . . . . . . . C C    
1866	. . . . . . . . . . . . . . . C C    
1867	. . . . . . . . . . . . . . . C C    
1868	. . . . . . . . . . . . . . . C C    
1869	. . . . . . . . . . . . . . . T T    
1870	. . . . . . . . . . . . . . . C C    
1871	. . . . . . . . . . . . . . . G G    
1872	. . . . . . . . . . . . . . . C C    
1873	. . . . . . . . . . . . . . . T T    
1874	. . . . . . . . . . . . . . . G G    
1875	. . . . . . . . . . . . . . . C C    
1876	. . . . . . . . . . . . . . . T T    
1877	. . . . . . . . . . . . . . . C C    
1878	. . . . . . . . . . . . . . . T T    
1879	. . . . . . . . . . . . . . . G G    
1880	. . . . . . . . . . . . . . . C C    
1881	. . . . . . . . . . . . . . . T T    
1882	. . . . . . . . . . . . . . . T T    
1883	. . . . . . . . . . . . . . . G G    
1884	. . . . . . . . . . . . . . . G G    
1885	. . . . . . . . . . . . . . . T T    
1886	. . . . . . . . . . . . . . . G G    
1887	. . . . . . . . . . . . . . . A A    
1888	. . . . . . . . . . . . . . . G G    
1889	. . . . . . . . . . . . . . . . G    
1890	. . . . . . . . . . . . . . . . G    
1891	. . . . . . . . . . . . . . . . G    
1892	. . . . . . . . . . . . . . . . G    
1893	. . . . . . . . . . . . . . . . A    
1894	. . . . . . . . . . . . . . . . G    
1895	. . . . . . . . . . . . . . . . C    
1896	. . . . . . . . . . . . . . . . G    
1897	. . . . . . . . . . . . . . . . T    
1898	. . . . . . . . . . . . . . . . T    
1899	. . . . . . . . . . . . . . . . A    
1900	. . . . . . . . . . . . . . . . G    
1901	. . . . . . . . . . . . . . . . G    
1902	. . . . . . . . . . . . . . . . G    
1903	. . . . . . . . . . . . . . . . A    
1904	. . . . . . . . . . . . . . . . T    
1905	. . . . . . . . . . . . . . . . G    
1906	. . . . . . . . . . . . . . . . C    
1907	. . . . . . . . . . . . . . . . A    
1908	. . . . . . . . . . . . . . . . T    
1909	. . . . . . . . . . . . . . . . C    
1910	. . . . . . . . . . . . . . . . G    
1911	. . . . . . . . . . . . . . . . C    
1912	. . . . . . . . . . . . . . . . C    
1913	. . . . . . . . . . . . . . . . C    
1914	. . . . . . . . . . . . . . . . C    
1915	. . . . . . . . . . . . . . . . T    
1916	. . . . . . . . . . . . . . . . A    
1917	. . . . . . . . . . . . . . . . C    
1918	. . . . . . . . . . . . . . . . C    
1919	. . . . . . . . . . . . . . . . G    
1920	. . . . . . . . . . . . . . . . A    
1921	. . . . . . . . . . . . . . . . T    
1922	. . . . . . . . . . . . . . . . G    
1923	. . . . . . . . . . . . . . . . C    
1924	. . . . . . . . . . . . . . . . C    
1925	. . . . . . . . . . . . . . . . C    
1926	. . . . . . . . . . . . . . . . T    
1927	. . . . . . . . . . . . . . . . T    
1928	. . . . . . . . . . . . . . . . A    
1929	. . . . . . . . . . . . . . . . A    
1930	. . . . . . . . . . . . . . . . G    
1931	. . . . . . . . . . . . . . . . C
```
